# Travelling-Wave Electrophoresis, Electro-Hydrodynamics, Electro-Rotation, and Symmetry-Breaking of a Polarizable Dimer in Non-Uniform Fields

**DOI:** 10.3390/mi13081173

**Published:** 2022-07-25

**Authors:** Touvia Miloh, Eldad J. Avital

**Affiliations:** 1 School of Mechanical Engineering, Tel Aviv University, Tel Aviv 69978, Israel; 2 School of Engineering and Materials Science, Queen Mary University of London, London E1 4NS, UK; e.avital@qmul.ac.uk

**Keywords:** induced-charge electroosmosis, electrophoresis, electro-rotation, electro-hydrodynamics, polarization, travelling wave, Janus mobility, dimer (touching spheres), tangent-sphere coordinates

## Abstract

A theoretical framework is presented for calculating the polarization, electro-rotation, travelling-wave dielectrophoresis, electro-hydrodynamics and induced-charge electroosmotic flow fields around a freely suspended conducting dimer (two touching spheres) exposed to non-uniform direct current (DC) or alternating current (AC) electric fields. The analysis is based on employing the classical (linearized) Poisson–Nernst–Planck (PNP) formulation under the standard linearized ‘weak-field’ assumption and using the tangent-sphere coordinate system. Explicit expressions are first derived for the axisymmetric AC electric potential governed by the Robin (mixed) boundary condition applied on the dimer surface depending on the resistance–capacitance circuit (RC) forcing frequency. Dimer electro-rotation due to two orthogonal (out-of-phase) uniform AC fields and the corresponding mobility problem of a polarizable dimer exposed to a travelling-wave electric excitation are also analyzed. We present an explicit solution for the non-linear induced-charge electroosmotic (ICEO) flow problem of a free polarized dimer in terms of the corresponding Stokes stream function determined by the Helmholtz–Smoluchowski velocity slip. Next, we demonstrate how the same framework can be used to obtain an exact solution for the electro-hydrodynamic (EHD) problem of a polarizable sphere lying next to a conducting planar electrode. Finally, we present a new solution for the induced-charge mobility of a Janus dimer composed of two fused spherical colloids, one perfectly conducting and one dielectrically coated. So far, most of the available electrokinetic theoretical studies involving polarizable nano/micro shapes dealt with convex configurations (e.g., spheres, spheroids, ellipsoids) and as such the newly obtained electrostatic AC solution for a dimer provides a useful extension for similar concave colloids and engineered particles.

## 1. Introduction

An induced-charge electroosmosis (ICEO) is a non-linear physical phenomenon by which an uncharged or a neutrally charged polarizable (conducting) colloid which is suspended in an electrolyte engenders a fluid motion around its surface due to an ambient electric field [1,2,3]. The electric field can be spatially uniform or non-uniform, steady (DC) or time-dependent (AC) and, when being applied, it changes the charge density within the electric double layer (EDL) surrounding the particle. The potential thus induced on the polarized particle is proportional to the applied field and the Helmholtz–Smoluchowski (HS) surface slip velocity is quadratic in the amplitude of the electric forcing [2]. For some special simply connected orthotropic 3D shapes (i.e., spheres, spheroids, and tri-axial ellipsoids) [4], it is possible under the ’weak-field’ assumption to find by linearizing the governing Poisson-Nernst-Planck (PNP) equations explicit expressions for the induced-charge electrophoretic (ICEP) mobility of the colloid, subject to DC or AC excitations and finite EDL thickness [5,6]. Similar analytic expressions can also be obtained for the quadrupolar ICEO flow field that is induced around a symmetric stationary free particle, exhibiting fluid pumping along the direction of the field and fluid ejection in the normal direction [7]. A recent attempt to extend the analysis for some non-convex geometries which are not simply connected, such as horn (closed) toroidal shapes (resembling blood cells) is presented in [8]. A further theoretical generalization is provided here by considering the common dimer configuration, consisting of two touching (fused) spherical particles. For simplicity, we assume that the two colloids are of the same size and provide analytic solutions for some practical electrokinetic dimer cases by using the R-separable ‘tangent-sphere’ coordinate system [9].

The special arrangement of two (dimer) or more (chain) touching spherical particles often occurs in many branches of mathematical physics and nanotechnology, such as electrostatic [10,11,12,13,14,15,16,17,18,19,20,21] and optics [22,23,24,25]. The tangent-sphere coordinate system can be effectively used for analytically tackling some related problems involving particle-wall interactions in various electrokinetic [26,27,28,29,30,31,32,33,34,35], heat transfer [36,37,38], inviscid [39,40,41,42,43], and viscous [44,45,46,47,48,49,50] flow scenarios. Note that the corresponding tangent-sphere formulation can also be used as the leading-order (‘outer’) near-contact solution of a sphere lying next to an isothermal wall or a planar electrode, both for DC and AC (high-frequency) electrokinetic problems [32,34,36]. As far as we understand, the quadratic induced-charge electrophoretic (ICEP) and the related electro-hydrodynamic (EHD) problems of two fused spherical colloids which are subjected to non-uniform AC electric ambient fields, including some pertinent symmetry-breaking (Janus) aspects, have not been addressed before and thus this work can be considered as a first attempt in this direction.

The structure of the paper is as follows: In Section 2, we present an analytic solution for the ‘standard’ ICEO problem of a dimer composed of two fused conducting spherical colloids which are exposed to a spatially uniform AC axial ambient electric field. The analysis is performed under the ‘weak’ field and thin EDL assumptions, linearizing the PNP equations and applying the Robin (mixed) electrostatic boundary condition on the dimer surface in terms of the rivalling RC frequency [51,52]. The far-field dipole and the dimer polarization are also found in the course of the analysis. In Section 3, we address the corresponding electro-rotation (ROT) problem of a dimer as a result of applying two orthogonal uniform AC fields which are out of phase along the longitudinal and transverse directions. Explicit expressions are thus provided for the incited angular velocities of the dimer, by assuming a low-Reynolds (creeping) flow. Next, we consider in Section 4 the case of a travelling-wave electrophoresis whereby applying a non-uniform axial AC electric field results in a finite mobility of the dimer. General expressions for the phoretic velocity are given in terms of the forcing frequency and amplitude of the applied field, which are shown to vanish for the special case when the ambient field is spatially uniform.

In Section 5, we address the ICEO flow problem around a stationary dimer under a uniform field and obtain a closed-form solution for the velocity field in terms of the Stokes stream function by enforcing the HS velocity slip condition. The related problem of a freely suspended conducting sphere placed near a planar electrode is then considered in Section 6 and the corresponding EHD velocity field is explicitly solved by providing an analytic expression for the Stokes stream function. Finally, we analyze in Section 7 a typical case of a dimer symmetry-breaking, by considering a Janus arrangement of two spheres (one metallic and one dielectric) that are subjected to a uniform ambient field in the direction along the centers. It is demonstrated that as a result of the metallo-dielectric Janus asymmetry, the dimer will acquire a finite phoretic velocity (in contrast to the homogeneous case) in the direction of the metallic sphere. We conclude with some discussions in Section 8. A list of symbols and abbreviations appears after Section 8.

## 2. Polarization

**Figure 1:**
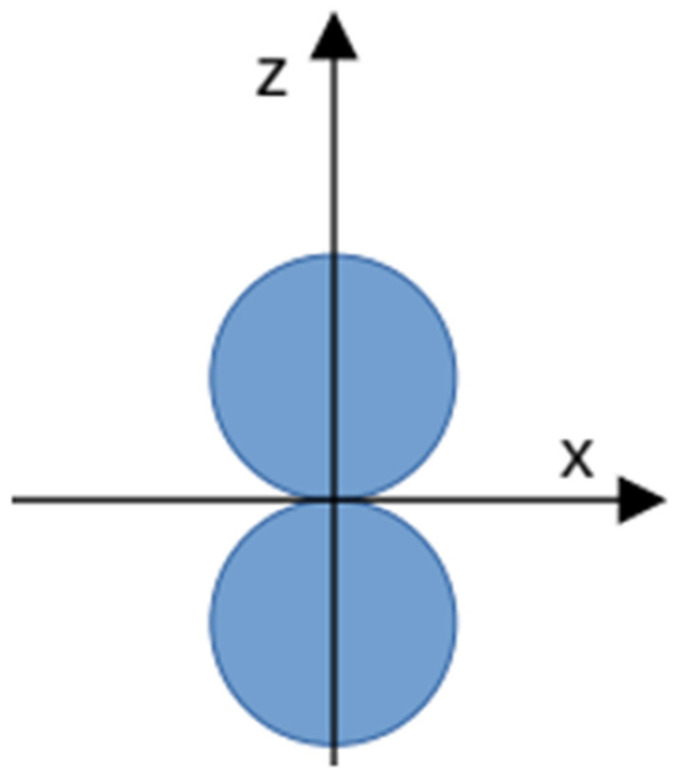
Schematic description of the problem of a dimer that is composed of two geometrically identical spheres in free space as in Section 2, Section 3, Section 4 and Section 5 and Section 7. The dimer is subjected to: a uniform AC electric field acting in the z direction (Section 2, Section 3, Section 4 and Section 5 and Section 7), a uniform AC electric field acting in the x direction (Section 3), and a non-homogeneous axisymmetric travelling wave propagating along the z direction (Section 4). The two spheres are identically conductive in Section 2, Section 3, Section 4 and Section 5, and in Section 7 the lower sphere is coated by a thin dielectric layer.

It is convenient to express the dimer (two touching spheres) geometry shown in Figure 1 in terms of a semi-separable curvilinear three-dimensional (3D) orthogonal tangent-sphere coordinate system $\left(\mu ,\upsilon ,\varphi \right)$ [9], which is related to the Cartesian system (*x*, *y*, *z*) by:

(1) $$z=\frac{\upsilon }{\mu^{2}+\upsilon^{2}};\;\;\;\;x+iy=\frac{\mu e^{i\varphi }}{\mu^{2}+\upsilon^{2}}$$

The Cartesian coordinates are normalized by 2a (sphere diameter), $\mu \in \left[0,\infty \right]$, $\upsilon \in \left[-\infty ,\infty \right]$, $\varphi \in \left[0,2\pi \right]$, and υ=±1 represent the surface of each sphere. At the origin of the tangent-sphere coordinate system, both μ and υ tend to infinity and in the far- field they approach zero.

A general (‘external’) harmonic function, which vanishes at infinity, can be written as (see [9], p. 104):

(2) χμ,υ,φ=μ2+υ21/2Re∑n=0∞einφ∫−∞∞AnsJnμseυsds

where $A_{n}\left(s\right)$ are generally complex functions such that the above integral is convergent and Re means the real part. An axisymmetric field (*n* = 0), which is antisymmetric with respect to υ can be written as:

(3) $$\chi_{0}\left(\mu ,\upsilon \right)={\left(\mu^{2}+\upsilon^{2}\right)}^{1/2}\int_{0}^{\infty }A_{0}\left(s\right)J_{0}\left(\mu s\right)\frac{sinh\left(s\upsilon \right)}{s*cosh\left(s\right)}ds.$$

Making use of the following identity (see [53], 6.611.1):

(4) $$\frac{1}{{\left(\mu^{2}+\upsilon^{2}\right)}^{1/2}}=\int_{0}^{\infty }J_{0}\left(\mu s\right)e^{-s\upsilon }ds\;$$

one immediately finds from Equation (1) that

(5) $$z={\left(\mu^{2}+\upsilon^{2}\right)}^{1/2}\int_{0}^{\infty }sJ_{0}\left(\mu s\right)e^{-s\upsilon }ds.$$

Let us next consider an ideally polarizable (conducting) dimer that is exposed to a uniform ambient AC electric field (unit amplitude) acting along the z axis of symmetry. A general expression for the AC potential $\phi_{o}\left(\mu ,\upsilon ,t\right)$ can then be written in terms of its phase as Reϕ0μ,υe−iωt, where t denotes time, ω is the forcing frequency, and $\phi_{0}\left(\mu ,\upsilon \right)=-z+\chi_{0}\left(\mu ,\upsilon \right)$.

When the polarized (initially unchanged) dimer is freely suspended in an electrolyte, its surface is generally screened by an electric double layer (EDL) of a nano-metric size $\lambda_{0}$, so that the boundary conditions governing the surface potential is of a Robin (mixed) type [51], i.e.,

(6) $$\frac{\partial \phi_{0}\left(\mu ,\upsilon \right)}{\partial n}=-i\Omega \phi_{0}\left(\mu ,\upsilon \right)\;\mathrm{on}\;\upsilon =1;\;\;\;\;\Omega =\frac{2a\omega \lambda_{0}}{D}$$

where *D* represents the diffusivity of the symmetric monovalent electrolyte and Ω denotes the common RC dimensionless frequency [1]. The normal derivative in Equation (6) can be also written as $\partial /\partial n=1/h_{\upsilon }\partial /\partial \upsilon$, where $h_{\upsilon }=1/\left(\mu^{2}+\upsilon^{2}\right)$ denotes the corresponding metric coefficient.

Thus, substituting Equations (3) and (5) into Equation (6) and following the same procedure as in [41,43] leads to:

(7) $$\left[\frac{\partial }{\partial \upsilon }+i{\left(\mu^{2}+\upsilon^{2}\right)}^{-1}\Omega \right]\left\{\frac{-\upsilon }{\mu^{2}+\upsilon^{2}}+{\left(\mu^{2}+\upsilon^{2}\right)}^{1/2}\int_{0}^{\infty }A_{0}\left(s\right)J_{0}\left(\mu s\right)\frac{sinh\left(s\upsilon \right)}{s*cosh\left(s\right)}ds\right\}=0$$

to be applied on υ=1. Finally, substituting Equation (4) in Equation (7) results in the following second-order inhomogeneous ordinary differential equation (ODE) for the coefficient A_0_(s):

(8) $$\frac{d^{2}A_{0}\left(s\right)}{ds^{2}}-\frac{1}{s}\frac{dA_{0}\left(s\right)}{ds}-\left[1+\left(1+i\Omega \right)\frac{tanh\left(s\right)}{s}-\frac{1}{s^{2}}\right]A_{0}\left(s\right)=\left[\left(2-i\Omega \right)s-1\right]e^{-s}$$

The solubility condition of Equation (3) implies that *A*_0_(*s*) must vanish both for s→0 and s→∞.

An exact analytical solution of Equation (8) for Ω=0 (DC limit) has been given by [38] as:

(9) $$\underset{\Omega \to 0}{\lim A_{0}\left(s\right)}=\frac{1}{2}s*cosh\left(s\right)\left[log\left(2*cosh\left(s\right)\right)+s\left(tanh\left(s\right)-2\right)\right]$$

As far as we know, an exact analytical solution of Equation (8) for Ω≠0 is not known. Following the works of [41,43], who dealt with a similar ODE, a good approximation for the ‘exact’ solution of Equation (8) can be obtained by using the corresponding ‘asymptotic’ solution, namely for s→∞. Indeed, by letting s→∞ in Equation (8), one finds to leading- order:

(10) $$A_{0}\left(s,\Omega \right)≃\frac{s}{4+i\Omega }\left[\frac{2\left(1+i\Omega \right)}{2+i\Omega }-\left(2-i\Omega \right)s\right]e^{-s}$$

It is worth mentioning that expanding Equation (9) for large *s* yields $A_{0}\left(s\to \infty ,0\right)=\left(s/4\right)\left(1-2s\right)e^{-s}$ in agreement with Equation (10). In addition, note that letting Ω→∞ in Equation (8) renders $A_{0}\left(s,\Omega \to \infty \right)=s^{2}e^{-s}$, which is again in accordance with Equation (10). For small values of Ω the right-hand side of Equation (10) can be expressed as $A_{0}\left(s\right)+\left(i\Omega /4\right)s^{2}e^{-s}+O\left(\Omega^{2}\right)$ where $A_{0}\left(s\right)$ is explicitly given by Equation (9).

**Figure 2:**
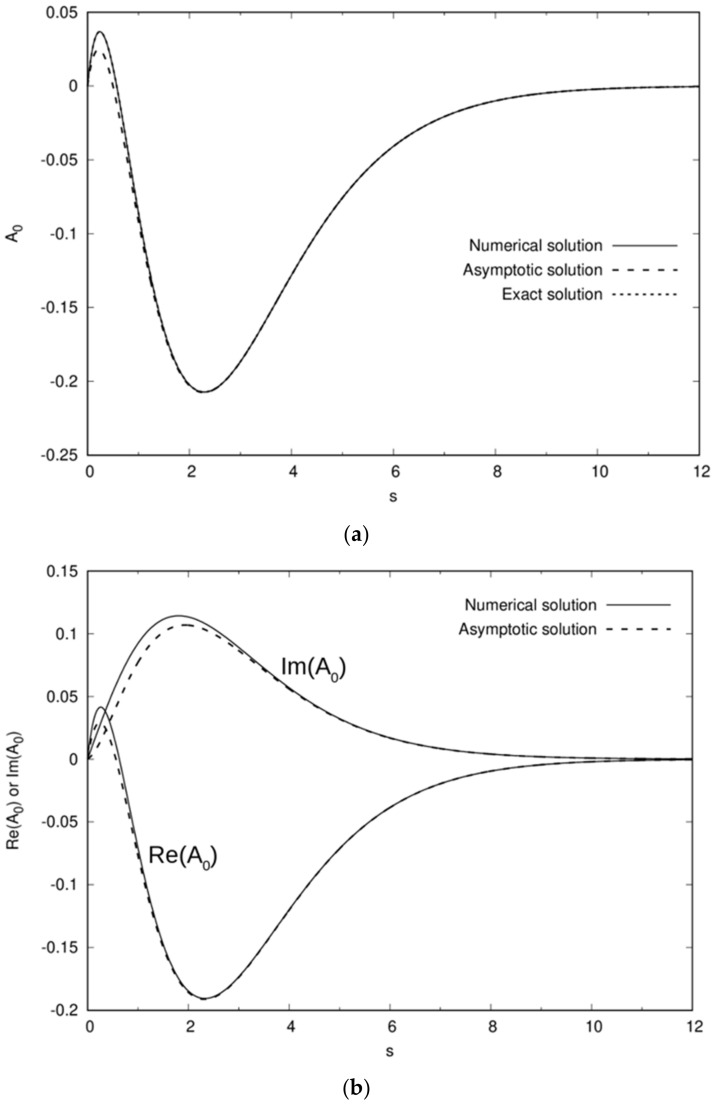

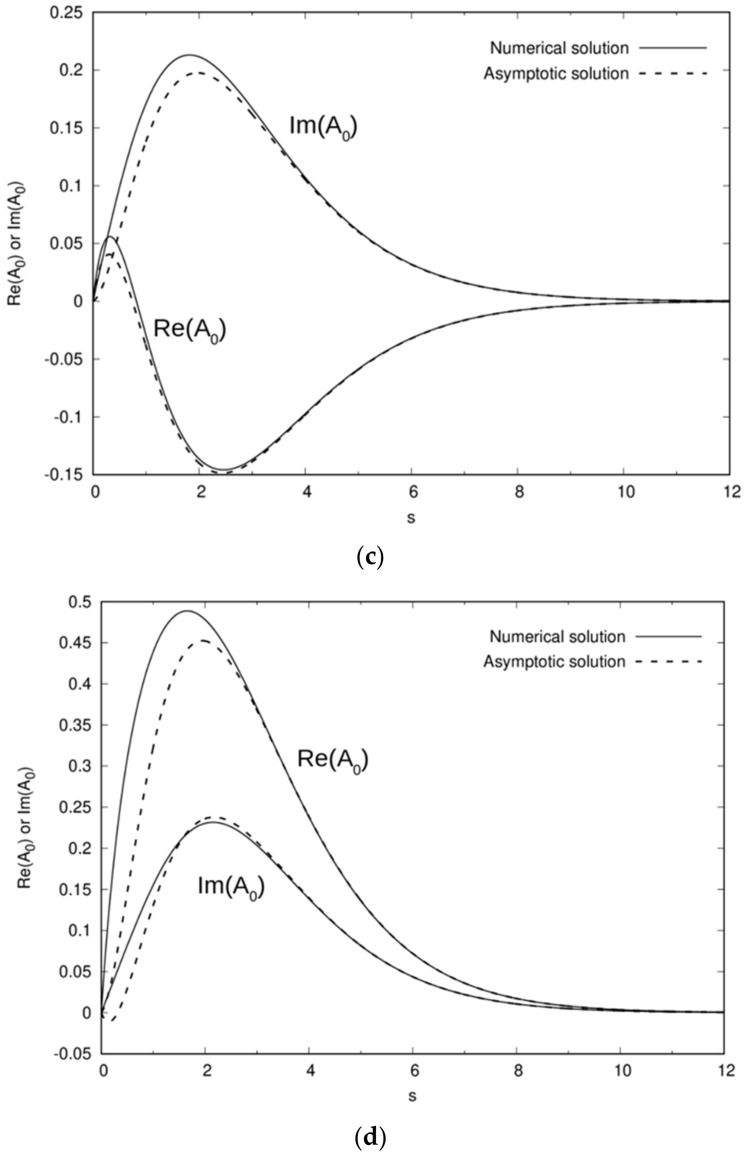
The solution of Equation (8) of Section 2 for (**a**) Ω=0, (**b**) Ω=0.5, (**c**) Ω=1, and (**d**) Ω=10, and where the exact solution of Ω=0 is given by Equation (9). The asymptotic solution is of Equation (10).

A full numerical solution of Equation (8) for Ω≠0 can be found by discretizing Equation (8) and using a second-order central finite difference scheme, yielding a tri-diagonal matrix that can be directly solved. A comparison between the numerical solution of both ReA0s,Ω and ImA0s,Ω with the asymptotic solution of Equation (10) where Im represents the imaginary part, generally shows good agreement (Figure 2). Note the excellent agreement obtained for Ω=0, as well as for relatively low frequencies, when comparing the numerical solution of Equation (8) with both the exact and asymptotic solutions given in Equations (9) and (10), respectively. It should also be noted that for large values of Ω, e.g., Ω=10, both the real and imaginary parts of $A_{0}\left(s,\Omega \right)$ are in the same phase whereas for Ω=0.5 or Ω=1 they are largely of opposite sign. This is due to a delayed change in the behaviour of the real part of $A_{0}\left(s,\Omega \right)$ becoming fully positive whereas the imaginary part is already fully positive at Ω=0.5.

Once the coefficients $A_{0}\left(s,\Omega \right)$ are found, one can determine the corresponding polarizability of a dimer by examining the far-field behaviour of Equation (3) along the *z*-axis $\left(\mu =0\right)$ as υ→0. Thus, by replacing $sinh\left(s\upsilon \right)$ in Equation (3) to leading- order of sυ and noting that $\upsilon^{2}\to 1/z^{2}$, one obtains the following expression for the dipole term d_0_ (normalized with respect to the dimensionless volume π/3 of the dimer [31]):

(11) $$\underset{\upsilon \to 0}{\lim\chi_{0}\left(0,\upsilon \right)}=\upsilon^{2}\int_{0}^{\infty }\frac{A_{0}\left(s\right)}{cosh\left(s\right)}ds,\;\;\;\;\;\;d_{0}\left(0\right)=-\int_{0}^{\infty }\frac{A_{0}\left(s\right)}{cosh\left(s\right)}ds$$

Let us first evaluate the corresponding far-field dipole *d*_0_(0) defined in Equation (11) by substituting the exact solution given in Equation (9), which renders (see [53], 3.523.3 & 3.557.3):

(12) $$d_{0}\left(0\right)=-\frac{1}{2}\int_{0}^{\infty }s\left\{ln[2cosh\left(s\right)[+s\left[tanh\left(s\right)-2\right]\right\}ds=\frac{1}{2}\int_{0}^{\infty }\left[\frac{s^{3}}{2cosh^{2}\left(s\right)}-\frac{s^{2}e^{-s}}{cosh\left(s\right)}\right]ds\;=\frac{3}{32}\zeta \left(3\right),$$

where $\zeta \left(3\right)$ denotes the Euler–Riemann zeta function $\left(\zeta \left(3\right)=1.202\ldots \right)$. Note that Equation (12) coincides with the longitudinal resistivity parameter, corresponding to two touching insulating identical spheres [38], obtained in the context of a dimer’s heat conduction and effective conductivity. Finally, we provide below an approximate solution for the frequency-dependent dipole term by substituting the solution of Equation (10) into Equation (11) which renders (see [53], 3.552.3):

(13) $$d_{0}\left(\Omega \right)=\frac{3}{8\left(4+i\Omega \right)}\left[\zeta \left(3\right)\left(2-i\Omega \right)-\frac{4\left(1+i\Omega \right)}{3\left(2+i\Omega \right)}\zeta \left(2\right)\right]$$

The above ‘asymptotic’ approximation for the AC dipole-term, may be also compared against the ‘exact’ value given in Equation (12) in the DC limit.

## 3. Electro-Rotation

Let us next consider the case of a uniform AC transverse forcing (acting in the *x* direction), where the corresponding asymmetric total ‘outer’ field (see Equation (2)) is now given by:

(14) $$\phi_{1}\left(\mu ,\upsilon ,\varphi \right)=-\frac{\mu cos\varphi }{\mu^{2}+\upsilon^{2}}+{\left(\mu^{2}+\upsilon^{2}\right)}^{1/2}cos\varphi \int_{0}^{\infty }A_{1}\left(s\right)J_{1}\left(\mu s\right)\frac{cosh\left(s\upsilon \right)}{s*sinh\left(s\right)}ds$$

The coefficients *A*_1_(*s*) in Equation (14) (symmetric with respect to υ), can be found in a similar manner to Equation (6) by enforcing the Robin boundary condition $\partial \phi_{1}/\partial \upsilon =-i\left(\mu^{2}+\upsilon^{2}\right)\Omega \phi_{1}$ on υ=1, resulting in the following second-order inhomogeneous ODE for *A*_1_(*s*):

(15) $$\frac{d^{2}A_{1}\left(s\right)}{ds^{2}}-\frac{1}{s}\frac{dA_{1}\left(s\right)}{ds}-\left[1+\left(1+i\Omega \right)\frac{coth\left(s\right)}{s}\right]A_{1}\left(s\right)=\left(2-i\Omega \right)se^{-s}$$

Equation (15) (compared to its ‘axisymmetric’ version of Equation (8)), is derived by using the relations below for the Bessel function (obtained from Equation (4)):

(16) $$\frac{\mu }{{\left(\mu^{2}+\upsilon^{2}\right)}^{3/2}}=\int_{0}^{\infty }sJ_{1}\left(\mu s\right)e^{-s\upsilon }ds;\;\;\;\;\left[\mu^{2}s^{2}+s\frac{d}{ds}\left(s\frac{d}{ds}\right)-1\right]J_{1}\left(\mu s\right)=0$$

It is interesting to note that unlike Equation (8), an exact solution of Equation (15) is not available even in the DC limit $\left(\Omega =0\right)$. Nevertheless, an asymptotic-type solution of Equation (15) can be obtained in a similar manner to Equation (10), by letting s→∞ which renders:

(17) $$A_{1}\left(s,\Omega \right)≃-s^{2}e^{-s}\frac{2-i\Omega }{4+i\Omega }+O\left(e^{-s}\right)$$

Thus, it appears that at least to a leading -order, $A_{0}\left(s,\Omega \right)=A_{1}\left(s,\Omega \right)$, as evidenced by obtaining the asymptotic limits of both Equations (8) and (15). In addition, note that the DC leading-order solution $A_{1}\left(s,0\right)=-s^{2}e^{-s}/2$ found for s→∞ from Equation (17) fully agrees with the DC asymptotic solutions obtained by [41,43].

The far-field dipole (along the *x*-axis) of Equation (14), normalized by the dimer non-dimensional volume π/3 [31], can again be found by setting υ=0 and letting μ→0, which renders

(18) $$\underset{\mu \to 0}{\lim\phi_{1}\;\left(0,\mu \right)}≃\frac{1}{2}\mu^{2}cos\varphi \int_{0}^{\infty }\frac{A_{1}\left(s\right)}{sinh\left(s\right)}ds,\;\;\;\;d_{1}\left(\Omega \right)=-\frac{1}{2}\int_{0}^{\infty }\frac{A_{1}\left(s,\Omega \right)}{sinh\left(s\right)}ds$$

since on *z* = 0, $\upsilon =0\;\mathrm{and}\;\mu^{2}={\left(x^{2}+y^{2}\right)}^{-1}$. Substituting Equation (17) in Equation (18) finally leads to the following asymptotic expression for the dispersion of the transverse frequency-dependent far-field dipole (see [53], 3.552.1):

(19) $$d_{1}\left(\Omega \right)=3\left(\frac{2-i\Omega }{4+i\Omega }\right)\zeta \left(3\right),\;$$

Electro-rotation (ROT) [6] of a dimer about its axis of symmetry can be achieved by applying two orthogonal (out-of-phase) uniform AC electric fields (of the same amplitude E_0_) acting in the x and y directions, such that the total field is given by:

(20) $$\vec{E}\left(t\right)=E_{0}\left({\hat{e}}_{x}-i\;{\hat{e}}_{y}\right)e^{-i\omega t}$$

where $\left({\hat{e}}_{x},{\hat{e}}_{y},{\hat{e}}_{z}\right)\;$ are the corresponding unit vectors along the (*x*, *y*, *z*) directions, respectively. The electrostatic (time-averaged) torque acting on the dimer can then be written in term of its effective dipole ${\vec{d}}_{eff}$ as [54]:

(21) $$\vec{\tau }=\frac{1}{2}\left({\vec{d}}_{eff}\times {\vec{E}}^{*}\right),$$

where the superscript (*) denotes the complex conjugate. Due to the axisymmetry of the dimer, the dimensional effective dipole is defined here using Equation (19) as ${\vec{d}}_{eff}=\epsilon E_{0}a^{3}d_{1}\left(\Omega \right)\left({\hat{e}}_{x}-i{\hat{e}}_{y}\right)e^{-i\omega t}$, where ϵ denotes the dimer’s permittivity. Substituting this expression for the effective dipole together with Equation (20) in Equation (21), taking the average over a single period, results in:

(22) τ→Ω=ϵE02a3Imd1Ωe^z=−9ϵE02a3ζ3Ω16+Ω2e^z.

Following [48,49], we recall that the resisting Stokes torque of a steady rotation of two touching equal spheres about their axis of symmetry, can be expressed as ${\vec{\tau }}_{s}=-12\pi \eta a^{3}{\dot{\Theta }}_{z}\zeta \left(3\right){\hat{e}}_{z}$, where ${\dot{\Theta }}_{\;}^{\;}$ is the corresponding angular velocity and η represents the dynamic viscosity of the solvent. Ignoring inertia effects and letting $\vec{\tau }≃{\vec{\tau }}_{s}$, one gets the following explicit solutions for the ROT angular velocity (mobility) of a dimer:

(23) $${\dot{\Theta }}_{z}=\frac{3\epsilon E_{0}^{2}}{4\pi \eta }\cdot \frac{\Omega }{16+\Omega^{2}}.$$

Thus, the ROT spectra given in Equation (23) is of a Lorentzian type, it vanishes both for zero and infinitely large forcing frequencies and exhibits a peak at Ω=4, such that ${\dot{\Theta }}_{z,max}^{\;}=3\epsilon E_{0}^{2}/\left(32*\pi *\eta \right)$.

A similar analysis can be also conducted for the complimentary case of an ‘asymmetric’ rotation of a dimer, say a rotation about its transverse *x*-axis, by considering the following asymmetric AC ambient field $\vec{E}\left(t\right)=E_{0}\left({\hat{e}}_{y}-i{\hat{e}}_{z}\right)e^{-i\omega t}$ instead of Equation (20). The corresponding dimensional effective dipole moment is now given as ${\vec{d}}_{eff}=\epsilon E_{0}a^{3}\left(d_{1}{\hat{e}}_{y}-id_{0}{\hat{e}}_{z}\right)e^{-i\omega t}$, where $d_{0}\left(\Omega \right)$ and $d_{1}\left(\Omega \right)$ are defined in Equations (13) and (19), respectively, which finally renders $\vec{\tau }=\epsilon E_{0}^{2}a^{3}I_{m}\left[d_{0}\left(\Omega \right)+d_{1}\left(\Omega \right)\right]{\hat{e}}_{x}/2$. The viscous creeping torque experienced by a rotating dimer about its transverse axes (*x*, *y*). has been numerically computed in [45] as ${\vec{\tau }}_{s}=-3.740\cdot 8\pi \eta a^{3}{\dot{\Theta }}_{x}{\hat{e}}_{x}$. Thus, by letting ${\vec{\tau }}_{s}≃\vec{\tau }$ (ignoring inertia effects) and using Equations (13) and (19), one obtains:

(24) $${\dot{\Theta }}_{x}\left(\Omega \right)=\frac{-\epsilon E_{0}^{2}}{16\eta \pi \cdot 3.740}I_{m}\left[d_{0}\left(\Omega \right)+d_{1}\left(\Omega \right)\right]=\frac{3\epsilon E_{0}^{2}}{8\eta \pi \cdot 3.740}\cdot \frac{\Omega }{16+\Omega^{2}}\left[\frac{15}{4}\zeta \left(3\right)+\frac{2-\Omega^{2}}{4+\Omega^{2}}\zeta \left(2\right)\right],$$

Equation (24) provides the sought explicit (asymptotic) expression for the transverse ROT spectra of a dimer consisting of two equal fused spheres. It is given again in a Lorentzian form of compact support which vanishes both for zero and infinitely large forcing frequencies.

## 4. Traveling-Wave Electrophoresis

It is well-known that a polarizable dimer embedded in ‘unbounded’ solute, which is exposed to a uniform (DC or AC) axisymmetric field, remains stationary although an induced-charge (dipole-type) electroosmotic (ICEO) flow is generated around its surface [2]. Nevertheless, if the ambient electric field is spatially non-uniform, the dimer acquires a linear velocity (mobility) in the z direction due to dielectrophoresis (DEP). In this section we consider the more general case of a polarized dimer that is subjected to an arbitrary non-homogenous travelling-wave (TW) excitation [54], whereby the ambient axisymmetric electric forcing is expressed in cylindrical coordinates $\left(z,r=\sqrt{x^{2}+y^{2}}\right)$, as

(25) ΧTWz,r,k;t=−Re{ X¯TWz,r,ke−iωt},

where ${\bar{Χ}}_{TW}\left(z,r,k\right)=\left(E_{0}/k\right)e^{i\left(kz-\varphi \right)}I_{0}\left(kr\right)$ and *I*_0_ is the modified Bessel function of the first kind and zero order. The forcing reference amplitude is denoted by *E*_0_, *k* represents its wave number, φ is an arbitrary phase angle, and ω is the forcing frequency. The particular form of Equation (25) is selected so that under the long-wave approximation $\left(k\to 0,\varphi =\pi /2\right)$ one gets Re { Χ¯TWz,r,0}=E0z, representing a time-harmonic axisymmetric ambient uniform field.

Expanding ${\bar{Χ}}_{TW}\left(z,0,k\right)$ in a Taylor series in k on the *z*-axis (*r* = 0) renders:

(26) $${\bar{Χ}}_{tw}\left(z,0,k\right)=\sum_{n=0}^{\infty }C_{n}z^{n}+\mathrm{const},\;\;\;\;\;\;\;\;C_{n}=\frac{E_{0}{\left(ik\right)}^{n-1}}{n!}z^{n}e^{-i\varphi }.$$

However, we recall that a general axisymmetric harmonic function which is proportional to *z^n^* can be also expressed in term of a Legendre polynomial $P_{n}\left(\tilde{\eta }\right)$ as $R^{n}P_{n}\left(\tilde{\eta }\right)$, where *R*^2^ = *r*^2^ + *z*^2^ and $\tilde{\eta }=cos^{-1}\left(z/R\right)$. Note that on the *z*-axis (*r* = 0), *R* = *z* and $\tilde{\eta }=0$. Thus, the polynomial *z*^n^ can be considered as the limiting value of an axisymmetric harmonic function of (*z*, *r*) evaluated on *r* = 0. Hence, following Equations (1), (2), and (26), one gets for υ>0 and μ→0:

(27) $$\underset{\mu \to 0}{\lim}\left[R^{n}P_{n}\left(\tilde{\eta }\right)\right]=z^{n}=\frac{1}{\upsilon^{n}}=\underset{\mu \to 0}{\lim}\{\;{\left(\mu^{2}+\upsilon^{2}\right)}^{1/2}\int_{0}^{\infty }A_{0}^{\left(n\right)}\left(s\right)J_{0}\left(\mu s\right)e^{-\upsilon s}ds\}.$$

Finally, by virtue of the identity in Equation (4), one can deduce by repeated differentiations that $A_{0}^{\left(n\right)}=s^{n}/n!$, which reduces to Equation (5) for *n* = 1.

The total electric potential, including the ambient field of Equation (27) and the scattering field given in Equation (3), can then be expressed for odd n, as:

(28) $$\phi_{0}^{\left(n\right)}\left(\mu ,\upsilon \right)=-R^{n}P_{n}\left(\tilde{\eta }\right)+\chi^{\left(n\right)}\left(\mu ,\upsilon \right)={\left(\mu^{2}+\upsilon^{2}\right)}^{1/2}\int_{0}^{\infty }\left[A_{0}^{\left(n\right)}\left(s\right)\frac{sinh\left(\upsilon s\right)}{s*cosh\left(s\right)}-\frac{s^{n}}{n!}e^{-\upsilon s}\right]J_{0}\left(\mu s\right)ds.$$

A similar form to Equation (28) is also available for even values of n by simply replacing $sinh\left(\upsilon s\right)/cosh\left(s\right)$ with $cosh\left(\upsilon s\right)/sinh\left(s\right)$. The unknown coefficients $A_{0}^{\left(n\right)}\left(s\right)$ are then found by enforcing the Robin-type boundary conditions of Equation (6) on $\chi^{\left(n\right)}\left(\mu ,\upsilon \right)$, which is represented by the first integral on the right-hand side of Equation (28), resulting in the following differential equation (ODE) for $A_{0}^{\left(n\right)}\left(s\right)$ (n-odd):

(29) $$\frac{d^{2}A_{0}^{\left(n\right)}\left(s\right)}{ds^{2}}-\frac{1}{s}\frac{dA_{0}^{\left(n\right)}\left(s\right)}{ds}-\left[1+\left(1+i\Omega \right)\frac{tanh\left(s\right)}{s}-\frac{1}{s^{2}}\right]A_{0}^{\left(n\right)}\left(s\right)=\frac{s^{n}}{n!}\left[\left(1+\frac{1-i\Omega }{n}\right)s-n\right]e^{-s}.$$

The corresponding ODE for even values of *n* is obtained by replacing $tanh\left(s\right)$ in Equation (29) with $coth\left(s\right)$. Note that Equation (29) reduces to Equation (8) as expressed for *n* = 1. Equation (29) does not yield an exact solution; however, an approximate expression for $A_{0}^{\left(n\right)}\left(s\right)$ (any *n*), can be found in a similar manner to Equation (8), in Section 2, by considering the limit s→0 in Equation (29), leading to:

(30) $$A_{0}^{\left(n\right)}\left(s\right)=\frac{s^{n}}{\left(n-1\right)!\left(2n+2+i\Omega \right)}\left[\frac{2\left(1+i\Omega \right)}{2+i\Omega /n}-\left(2-\frac{i\Omega }{n}\right)s\right]e^{-s}+O\left(s^{n-1}e^{-n}\right),$$

which again reduces to Equation (10) for *n* = 1.

In order to find the far-field multipoles of $\chi^{n}\left(\mu ,\upsilon \right)$ in Equation (28) prevailing along the axis of symmetry r=0, z→∞, it is enough again to expand $sinh\left(\upsilon s\right)$ in a Taylor series for μ=0 and υ→0, resulting in

(31) $$\chi^{\left(n\right)}\left(0,\upsilon \to 0\right)∼\sum_{m=0}^{\infty }\frac{\upsilon^{2m+2}}{\left(2m+1\right)!}\int_{0}^{\infty }A_{0}^{\left(n\right)}\left(s\right)\frac{s^{2m}}{cosh\left(s\right)}ds.$$

Next, recalling that $\chi^{n}$ in Equation (31) can be also expanded along the axis of symmetry (z→∞), in terms of the far-field multipoles ${\tilde{d}}_{m}^{\left(n\right)}$ as:

(32) $$\chi^{\left(n\right)}\left(0,\upsilon \to 0\right)=\sum_{m=0}^{\infty }{\tilde{d}}_{m}^{\left(n\right)}\frac{d^{m}}{dz^{m}}\left(\frac{1}{z}\right)=\sum_{m=0}^{\infty }{\left(-1\right)}^{m}m!{\tilde{d}}_{m}^{\left(n\right)}\upsilon^{m+1},$$

implying that the corresponding multipoles of order *m* and odd *n,* are explicitly given by:

(33) $${\tilde{d}}_{2m+1}^{\left(n\right)}=\frac{-1}{{\left[\left(2m+1\right)!\right]}^{2}}\int_{0}^{\infty }A_{0}^{\left(n\right)}\left(s\right)\frac{s^{2m}}{cosh\left(s\right)}ds.$$

The steady-state (time-averaged) dielectrophoretic force component *F*_DEP,_ which is exerted on the dimer by the travelling-wave ambient field given in Equations (25) and (26), can then be expressed following [5,54] in terms of the above multipoles as:

(34) FDEP=2πRe∑n=0∞−1nCndnnn!dn+1dzn+1∑m=0∞Cm*zmz=0=−4πRe∑m=0∞m+1C2m+1C2m+2*d2m+12m+2,

where the TW amplitudes *C*_m,_ are defined in Equation (26). Note that only the odd-order (2*m* + 1) multipoles of Equation (33) contribute to the DEP force in Equation (34).

The higher-order multipole terms in Equation (33), can be next evaluated by substituting the leading-order asymptotic expression of $A_{0}^{\left(n\right)}\left(s\right)$ obtained in Equation (30) into Equation (33), resulting in:

(35) $${\tilde{d}}_{2m+1}^{\left(2m+2\right)}=\frac{4m+4-i\Omega }{\left(2m+2\right)!{\left[\left(2m+1\right)!\right]}^{2}\left[4m+6+i\Omega \right]}\int_{0}^{\infty }\frac{s^{4m+3}e^{-s}}{cosh\left(s\right)}ds.$$

The integral in Equation (35) can be evaluated analytically (see [53], 3.552.3) and thus one finds

(36) $${\tilde{d}}_{2m+1}^{\left(2m+2\right)}=\frac{4m+4-i\Omega }{4m+6+i\Omega }T\left(m\right),\;\;\;\;\;\;\;\;\;\;\;T\left(m\right)=\frac{2^{-\left(4m+3\right)}\left[1-2^{-\left(4m+3\right)}\right]\left(4m+3\right)!\zeta \left(4m+4\right)}{\left(2m+2\right)!{\left[\left(2m+1\right)!\right]}^{2}},$$

where $\zeta \left(n\right)$ denotes again the Riemann zeta function.

Finally, substituting $C_{m}$ defined in Equations (26) and (36) into Equation (34) leads to:

(37) $$F_{DEP}=4\pi E_{0}^{2}\Omega \sum_{n=0}^{\infty }\frac{\left(4m+3\right)}{\{\left(2m+1\right)!]2}\frac{k^{4m+1}T\left(m\right)}{\left[{\left(4m+6\right)}^{2}+\Omega^{2}\right]}.$$

Equation (37) is the sought expression for the axial travelling-wave dielectrophoretic (TWDEP) force acting on a polarizable dimer that is exposed to an arbitrary ambient non-uniform traveling-wave (TW) field prescribed by Equations (25) and (26), in terms of the dimensional RC frequency Ω defined in Equation (6) and the characteristic wave number k of the ambient field. The spectrum of Equation (37) is again of a Lorentzian type, vanishing both at Ω=0 (DC limit) and Ω→∞ (due to insufficient time for AC charging to take place over a single period), as well as for k→0 (infinitely long wave-length corresponding to a uniform field).

## 5. Induced-Charge Electroosmosis

Following the analysis presented in Section 2, we consider here the case of a freely suspended polarizable dimer, which is subjected to an axisymmetric AC uniform electric field. We are interested here in calculating the induced electroosmotic flow field prevailing around the colloid. Thus, the total electric field (of unit amplitude) incited in the surrounding electrolyte is given by $\phi_{0}\left(\mu ,\upsilon ,\Omega \right)=-z+\chi_{0}\left(\mu ,\upsilon ,\Omega \right)$, where the scattering potential $\chi_{0}\left(\mu ,\upsilon \right)$ is defined in Equation (3) in terms $A_{0}\left(s,\Omega \right)$. The coefficient $A_{0}\left(s,\Omega \right)$ is determined by applying the Robin-type boundary condition on the dimer surface $\left(\upsilon =\pm 1\right)$ in terms of the RC dimensionless frequency Ω defined in Equation (6) and is found by solving the inhomogeneous 2nd-order non-linear ODE given in Equation (8). As previously explained, for most practical numerical purposes, it is possible to use the one-term asymptotic approximation given in Equation (10), namely $A_{0}\left(s,\Omega \right)\approx \left[2\left(1+i\Omega \right)/\left(2+i\Omega \right)-\left(2-i\Omega \right)s\right]se^{-s}/\left(4+i\Omega \right)$. Hence, substituting this expression in Equation (3) and letting s→∞, leads to ([53], 6.623.2):

(38) $$\begin{array}{cc} \phi_{0}\left(\mu ,\pm 1,\Omega \right) & =\pm \left[-\frac{1}{1+\mu^{2}}+{\left(1+\mu^{2}\right)}^{1/2}\int_{0}^{\infty }A_{0}\left(s,\Omega \right)J_{0}\left(s\mu \right)\frac{ds}{s}\right]\\ & =\pm \frac{2}{4+i\Omega }\left[-\frac{3}{1+\mu^{2}}+\frac{1+i\Omega }{2+i\Omega }\right]. \end{array}$$

Next, assuming a ‘thin’ Debye layer (EDL) as compared to the dimer radius, the induced-charge distribution in the solvent engenders a HS slip velocity on the dimer surface [2,3]. For a perfectly conducting dimer, this surface velocity slip, when expressed in the present curvilinear coordinate system, can be written (dimensionless form) in terms of the potential $\phi_{0}\left(\mu ,\upsilon \right)$ as:

(39) $$U_{\mu }\left(\mu ,\pm 1,\Omega \right)=-\frac{sgn\left(\upsilon \right)}{2h_{\mu }}\frac{\partial {\left|\phi_{0}\left(\mu ,\pm 1,\Omega \right)\right|}^{2}}{\partial \mu }=\left[\frac{72\mu }{\left(16+\Omega^{2}\right){\left(1+\mu^{2}\right)}^{2}}-\frac{24\mu \left(2+\Omega^{2}\right)}{\left(16+\Omega^{2}\right)\left(4+\Omega^{2}\right)\left(1+\Omega^{2}\right)}\right]sgn\left(\upsilon \right).$$

Here $U_{\mu }\left(\mu ,\pm 1,\Omega \right)$ denotes the tangential velocity over the surface of the dimer and $h_{u}=h_{\upsilon }={\left(\mu^{2}+\upsilon^{2}\right)}^{-1}$ are the two metric coefficients of the curvilinear coordinates (tangent-sphere) system $\left(\mu ,\upsilon ,\varphi \right)$. It is clear that $U_{\mu }$ in Equation (39) is asymmetric with respect to υ (*z* = 0 plane) and a result the DEP force exerted on the dimer by a uniform field is null! Yet, the induced HS slip velocity on the colloid surface renders a dipole-type velocity field around the dimer which decays (as expected) away from the origin. The resulting induced-charge electroosmotic (ICEO) flow field in the solvent, is assumed to be governed by the Stokes momentum equation [2] (ignoring inertia) and thus under the present axisymmetric forcing, can be expressed in terms of a Stokes stream surface $\psi \left(\mu ,\upsilon \right)$, satisfying the following fourth-order PDE: $E^{4}\psi \left(\mu ,\upsilon \right)=0$, where:

(40) $$E^{2}\psi \left(\mu ,\upsilon \right)=\frac{1}{4}\mu \left(\mu^{2}+\upsilon^{2}\right)\left[\frac{\partial }{\partial \mu }\left(\frac{\mu^{2}+\upsilon^{2}}{\mu }\right)+\frac{\partial }{\partial \upsilon }\left(\frac{\mu^{2}+\upsilon^{2}}{\mu }\right)\right]\psi \left(\mu ,\upsilon \right).$$

A general solution of Equation (40) for a tangent-sphere coordinate system (containing four unknown coefficients), was given in [44]. Imposing the surface no-flux conditions $\psi \left(\mu ,\pm 1\right)=0$ and the asymmetry with respect to υ finally yields:

(41) $$\psi \left(\mu ,\upsilon ,\Omega \right)=\frac{\mu }{{\left(\mu^{2}+\upsilon^{2}\right)}^{3/2}}\int_{0}^{\infty }F\left(s,\Omega \right)\left[sinh\left(ns\right)-\upsilon *tanh\left(s\right)cosh\left(\upsilon s\right)\right]J_{1}\left(\mu s\right)ds,$$

where $F\left(s,\Omega \right)$ is yet to be determined.

The tangential velocity component, $U_{\mu }\left(\mu ,\pm 1,\Omega \right)$, can be next found following [44] directly from Equation (41) as:

(42) $$\begin{array}{c} U_{\mu }\left(\mu ,\pm 1,\Omega \right)=-\frac{{\left(1+\mu^{2}\right)}^{2}}{\mu }{\left.\frac{\partial \psi }{\partial \upsilon }\left(\mu ,\upsilon ,\Omega \right)\right|}_{\upsilon =\pm 1}\\ =-{\left(1+\mu^{2}\right)}^{1/2}\int_{0}^{\infty }F\left(s,\Omega \right)\left[sinh\left(s\right)-s/cosh\left(s\right)\right]J_{1}\left(\mu s\right)ds, \end{array}$$

Combining next Equations (39) and (42) yields:

(43) $$\int_{0}^{\infty }F\left(s,\Omega \right)\left[sinh\left(\upsilon s\right)-s/cosh\left(s\right)\right]J_{1}\left(\mu s\right)ds=-\frac{72\mu }{\left(16+\Omega^{2}\right){\left(1+\mu^{2}\right)}^{5/2}}+\frac{24\mu \left(2+\Omega^{2}\right)}{\left(16+\Omega^{2}\right)\left(4+\Omega^{2}\right){\left(1+\mu^{2}\right)}^{3/2}},$$

which can be inverted using ([53], 6.623.1), resulting in:

(44) $$F\left(s,\Omega \right)=\frac{24s^{2}e^{-s}\left[s-2\left(2+\Omega^{2}\right)/\left(4+\Omega^{2}\right)\right]}{\left(16+\Omega^{2}\right)\left[s*sinh\left(s\right)-s/cosh\left(s\right)\right]}.$$

Finally, substituting Equation (44) into Equation (41) provides the sought expression for the Stokes stream function governing the low-Reynolds (creeping) ICEO flow field about the polarized dimer. The corresponding curvilinear velocity components $\left(U_{\mu },U_{\upsilon }\right)$, induced in the electrolyte, can then be obtained by a proper differentiation of $\psi \left(\mu ,\upsilon ,\Omega \right)$, i.e., $U_{\mu }\left(\mu ,\upsilon ,\Omega \right)=-{\left(\mu^{2}+\upsilon^{2}\right)}^{2}/\mu \cdot \partial \psi /\partial \upsilon$ and $U_{\upsilon }\left(\mu ,\upsilon ,\Omega \right)={\left(\mu^{2}+\upsilon^{2}\right)}^{2}/\mu \cdot \partial \psi /\partial \mu$. Note also that $F\left(s,\Omega \right)$ is non-singular as s→0 and that the velocity field in the fluid decreases with the dimensionless RC frequency as ${\left(16+\Omega^{2}\right)}^{-1}$ (maximum velocity is attained at the DC limit Ω=0).

## 6. Electro-Hydrodynamics of a Particle Next to a Wall

**Figure 3:**
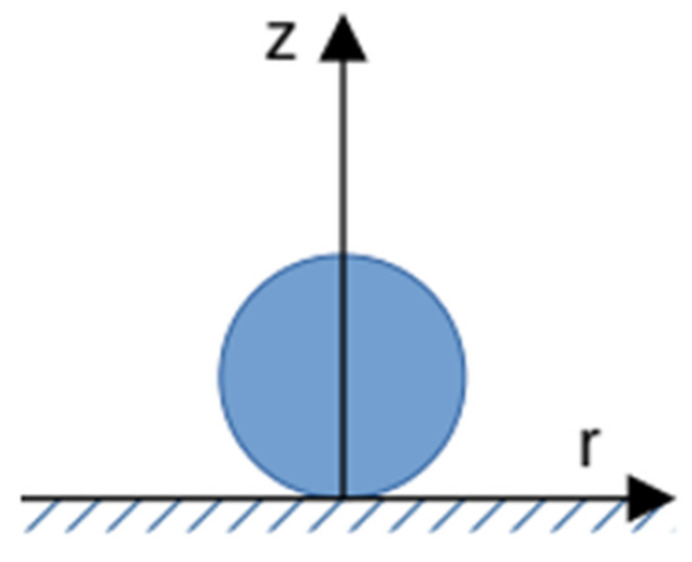
Schematic description of the problem in Section 6 of a spherical particle next to a wall (*z* = 0), which is subjected to a uniform DC electric field acting in the z direction.

Here, we demonstrate how the present methodology can be applied to obtain an analytic solution for the electro-hydrodynamic (EHD) flow field around a spherical colloid placed next to a conducting planar substrate (electrode) which is subjected to a uniform DC electric field excitatiin. In the case where the field is applied in a direction normal to the electrode, an explicit solution is found for the corresponding Stokes stream function. We consider a freely suspended initially uncharged polarizable particle (of unit diameter), lying next to a grounded planar electrode (z = 0), which is subjected to a uniform electric field $-E_{0}z$ as z→∞ (see Figure 3). The standard electrokinetic model combined with the thin EDL assumption (2), implies that the electric field in the solute $\phi \left(\mu ,\upsilon \right)$ is governed by the Laplace equation and satisfies

(45) $$\frac{1}{h_{\upsilon }}{\left.\frac{\partial \phi \left(\mu ,\upsilon \right)}{\partial \upsilon }\right|}_{\upsilon =0}=-E_{0},\;\;{\left.\frac{\partial \phi \left(\mu ,\upsilon \right)}{\partial \upsilon }\right|}_{\upsilon =1}=0.$$

Since the problem preserves axial symmetry with respect to the *z*-axis, a general solution for $\phi \left(\mu ,\upsilon \right)$ can then be expressed in terms of the tangent-sphere coordinates for υ≥0, as [9]:

(46) $$\frac{\phi \left(\mu ,\upsilon \right)}{E_{0}}=-\frac{\upsilon }{\mu^{2}+\upsilon^{2}}+{\left(\upsilon^{2}+\mu^{2}\right)}^{1/2}\int_{0}^{\infty }B\left(s\right)cosh\left(sv\right)J_{0}\left(\mu s\right)ds,$$

where B(s) is a coefficient to be determined. Note that Equation (46) automatically satisfies the boundary condition at υ=0 in Equation (45) on the plane *z* = 0. Enforcing next the Neumann boundary condition applied on the colloid surface $\left(\upsilon =1\right)$, given by Equation (45), leads to:

(47) $$\int_{0}^{\infty }B\left(s\right)cosh\left(sv\right)J_{0}\left(\mu s\right)ds+\left(1+\mu^{2}\right)\int_{0}^{\infty }sB\left(s\right)sinh\left(sv\right)J_{0}\left(\mu s\right)ds=\frac{1}{{\left(1+\mu^{2}\right)}^{1/2}}-\frac{2}{{\left(1+\mu^{2}\right)}^{3/2}}.$$

Recalling that the Bessel function satisfies $s\mu^{2}J_{0}\left(\mu s\right)=-d\left[sdJ_{0}\left(\mu s\right)/ds\right]/ds$ and using Equation (4), an integration by parts of Equation (47) results in the following inhomogeneous second-order ordinary differential equation:

(48) $$ssinh\left(s\right)\frac{d^{2}B\left(s\right)}{ds^{2}}+\left[sinh\left(s\right)+2scosh\left(s\right)\right]\frac{dB\left(s\right)}{ds}=\left(2s-1\right)e^{-s}.$$

Employing the general scheme outlined in ([55], p. 14), the first integral of Equation (48) which is finite for s→0, is given by:

(49) $$\frac{dB\left(s\right)}{ds}=1-coth\left(s\right)+\frac{s}{2sinh^{2}\left(s\right)}.$$

Thus, an explicit expression for *B*(*s*) (which vanishes for s→∞), can be found by integrating Equation (49), resulting in:

(50) $$B\left(s\right)=s-\frac{1}{2}s\;coth\left(s\right)-\frac{1}{2}ln\left[2\;sinh\left(s\right)\right].$$

such that $B\left(s\to 0\right)\to \left[ln\left(2s\right)+1\right]/2+O\left(s\right)$.

Once the electrostatic problem governed by Equations (46) and (48) has been solved, one can also consider the corresponding hydrodynamic problem. Following [44], a general explicit expression for the Stokes stream function of the creeping velocity field past an impermeable sphere in contact with a planar wall can be expressed in a tangent-sphere coordinate system in terms of four unknown coefficients $\left(\tilde{A},\;\tilde{B},\;\tilde{C},\;\tilde{D}\right)$ as:

(51) $$\Psi \left(\mu ,\upsilon \right)=\frac{\mu }{{\left(\mu^{2}+\upsilon^{2}\right)}^{3/2}}\int_{0}^{\infty }\left\{\left[\tilde{B}\left(s\right)+\upsilon \tilde{A}\left(s\right)\right]sinh\left(\upsilon s\right)+\left[\;D\;˜\left(s\right)+\upsilon \;C\;˜\left(s\right)\right]cosh\left(\upsilon s\right)J_{1}\left(\mu s\right)\right\}ds.$$

Since both the grounded electrode $\left(z=\upsilon =0\right)$ and particle $\left(\upsilon =1\right)$ are considered as stream surfaces, namely $\psi \left(\mu ,0\right)=\psi \left(\mu ,1\right)=0$, Equation (51) implies that $\tilde{D}\left(s\right)=0$. In addition, applying the vanishing velocity (no-slip) condition over the surface of the rigid polarizable colloid (see discussion in [30]) renders ${\left.\partial \psi \left(\mu ,\upsilon \right)/\partial \upsilon \right|}_{\upsilon =1}=0$. By virtue of the above three boundary conditions, one can express the general solution for the Stokes stream function given by Equation (51) in terms of a single coefficient $\tilde{C}\left(s\right)$ as:

(52) $$\psi \left(\mu ,\upsilon \right)=\frac{\mu }{{\left(\mu^{2}+\upsilon^{2}\right)}^{3/2}}\int_{0}^{\infty }\tilde{C}\left(s\right)\left[\frac{\upsilon sinh\left(s\left(1-\upsilon \right)\right)}{sinh\left(s\right)}-\frac{s\left(1-\upsilon \right)sinh\left(s\upsilon \right)}{sinh^{2}\left(s\right)}\right]J_{1}\left(\mu s\right)ds.$$

It is also important to note that Equation (52) is obtained by substituting:

(53) $$\tilde{A}\;\left(s\right)=\tilde{C}\left(s\right)\frac{s-sinh\left(s\right)cosh\left(s\right)}{sinh^{2}\left(s\right)},\tilde{B}\left(s\right)=-\tilde{C}\left(s\right)\frac{s}{sinh^{2}\left(s\right)},$$

in Equation (51).

In order to find the single coefficient $\tilde{C}\left(s\right)$ in Equation (52), we need to calculate the induced horizontal HS slip velocity on the planar electrode at the edge of the Debye layer (EDL), given by (see [2,30]) as:

(54) $$U_{z}\left(r,z=0\right)=U_{\upsilon }\left(\mu ,\upsilon =0\right)=-\frac{\epsilon \lambda_{0}E_{0}}{\eta }\left(\mu^{2}+\upsilon^{2}\right){\left.\frac{\partial \phi \left(\mu ,\upsilon \right)}{\partial \mu }\right|}_{\upsilon =0},.$$

where we recall that η  denotes the dynamic viscosity of the electrolyte. The EDL thickness is denoted by $\lambda_{0}$ and ε represents the relative permittivity of the solute. Expressed in terms of the Stokes stream function, the above slip velocity evaluated on the surface of the spherical colloid can be written (see [30]) as:

(55) $$U_{\upsilon }\left(\mu ,\upsilon =0\right)=-\frac{{\left(\mu^{2}+\upsilon^{2}\right)}^{2}}{\mu }{\left.\frac{\partial \psi \left(\mu ,\upsilon \right)}{\partial \upsilon }\right|}_{\upsilon =0}.$$

Thus, following Equations (54) and (55), one finds:

(56) $${\left.\frac{\partial \psi \left(\mu ,\upsilon \right)}{\partial \upsilon }\right|}_{\upsilon =0}=-\frac{\epsilon \lambda_{0}E_{0}}{\eta }\frac{\mu }{\mu^{2}+\upsilon^{2}}{\left.\frac{\partial \phi \left(\mu ,\upsilon \right)}{\partial \mu }\right|}_{\upsilon =0}.$$

The term on the left-hand side of Equation (56) can be readily found from Equation (52) as;

(57) $${\left.\frac{\partial \psi \left(\mu ,\upsilon \right)}{\partial \upsilon }\right|}_{\upsilon =0}=\frac{1}{\mu^{2}}\int_{0}^{\infty }\tilde{C}\left(s\right)\left[1-\frac{s^{2}}{sinh^{2}\left(s\right)}\right]J_{1}\left(\mu s\right)ds.$$

In a similar way, using Equation (46) we obtain:

(58) $${\left.\frac{\partial \phi \left(\mu ,\upsilon \right)}{\partial \mu }\right|}_{\upsilon =0}=\int_{0}^{\infty }\tilde{B}\left(s\right)J_{0}\left(\mu s\right)ds+\int_{0}^{\infty }s\tilde{B}\left(s\right)\frac{dJ_{0}\left(\mu s\right)}{ds}ds=-\int_{0}^{\infty }s\frac{d\tilde{B}\left(s\right)}{ds}J_{0}\left(\mu s\right)ds.$$

Next, integrating Equation (58) by parts and combining it with Equation (57), finally leads to:

(59) $$\tilde{C}\left(s\right)=-\frac{\epsilon E_{0}^{2}\lambda_{0}}{\eta }\frac{sd^{2}B\left(s\right)/ds^{2}}{1-{\left[s/sinh\left(s\right)\right]}^{2}}=-\frac{\epsilon E_{0}^{2}\lambda_{0}}{\eta }\frac{s\left[3/2-scoth\left(s\right)\right]}{sinh^{2}\left(s\right)-s^{2}}.$$

It should be noted that $\tilde{C}\left(s\right)$ vanishes for a large *s* but it is singular like $O\left(s^{-3}\right)$ for s→0. Nevertheless, we recall that the kernel in Equation (52) behaves like $\upsilon \left(1-\upsilon \right)s^{3}$ for small s, so that the integral in Equation (52) is well-defined. Substituting Equation (59) into Equation (52) provides the sought explicit expression for the Stokes stream function for the EHD velocity field generated around a polarized spherical colloid placed adjacent to a grounded electrode. Thus, the corresponding velocity components in the fluid domain, i.e., $\left(U_{\mu },U_{\upsilon }\right)=-{\left(\mu^{2}+\upsilon^{2}\right)}^{2}/\mu \cdot \left(\partial /\partial \upsilon ,-\partial /\partial \mu \right)\psi \left(\mu ,\upsilon \right)$, can be explicitly determined.

**Figure 4:**
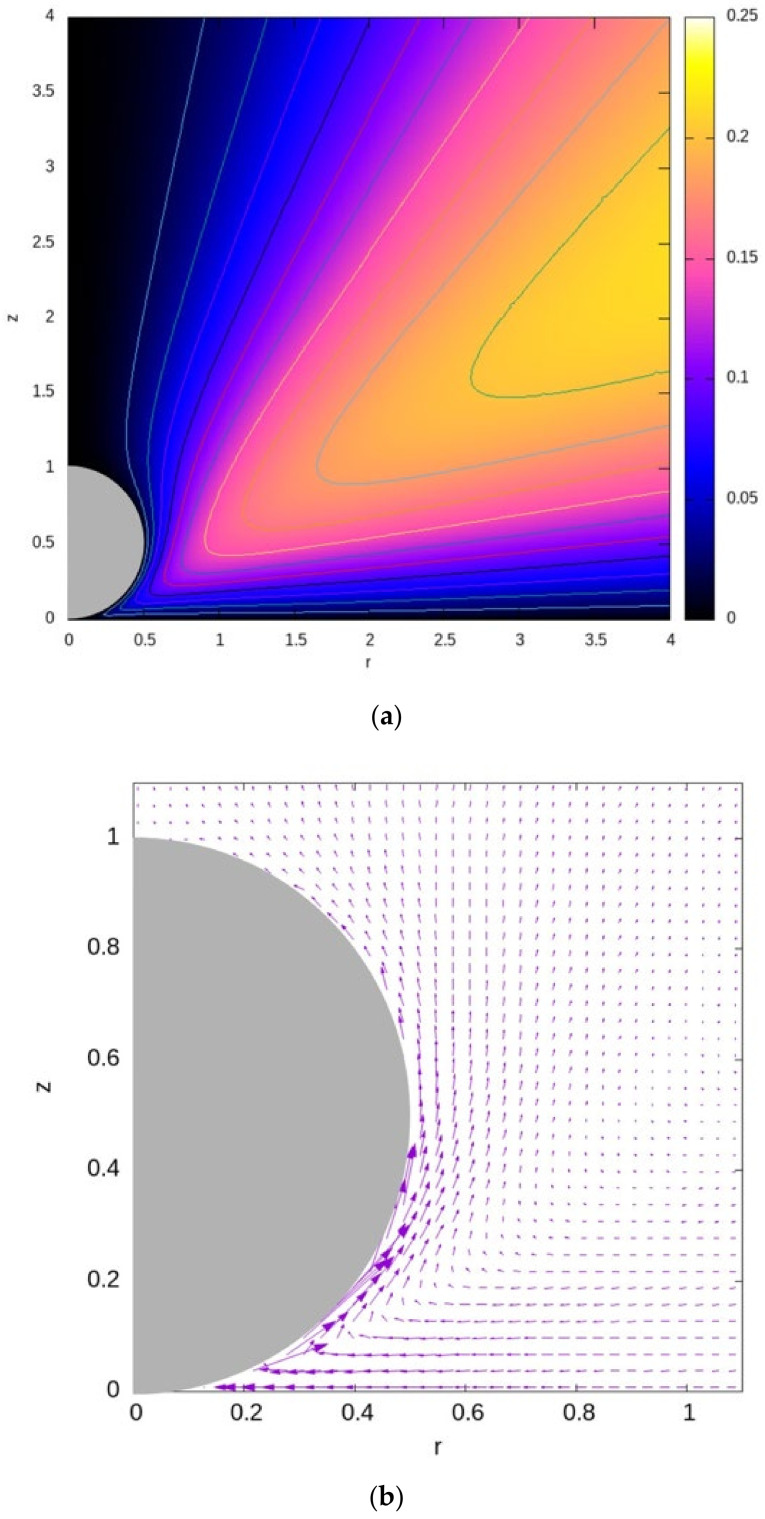
The (**a**) contours of the Stokes stream function and (**b**) velocity vectors around the spherical particle placed next to a wall at z = 0 of Section 6, and which is subjected to a uniform DC electric field acting in the z direction. The velocity-vector field modulus was adjusted for better viewing.

Solving the integral in the right-hand side of Equation (52) numerically leads to the contour plots of the Stokes stream function and the associated velocity fields depicted in Figure 4 (see also [30,34]). It is clearly seen that the highest velocity magnitude is observed along the lower surface of the sphere, where fluid is pushed into the gap between the sphere and the grounded wall/electrode, and then it rises up along the sphere. Hence, the EHD slip velocity of the surrounding fluid works to push the particle away from the wall (repulsion).

## 7. Induced-Charge Electrophoresis of a Janus Dimer

As a final demonstration of the above methodology, we consider a typical symmetry-breaking problem related to the mobility of two touching (fused) spherical colloidal particles of different surface properties that are exposed to an ambient electric field. For simplicity, we consider here the case of a dimer composed of two geometrically identical spheres, the upper one is taken to be perfectly conductive and the lower one is coated with a thin dielectric layer. The dimer is subjected to a uniform axisymmetric DC field acting along the z-axis (Figure 1). The effect of the coating is to suppress the induced-charge electroosmotic flow on the dielectric sphere [3] and due to material asymmetry, it leads to a finite induced-charge electrophoretic (ICEP) motion of the Janus dimer. It is important to note that the mobility of the Janus dimer arises from material symmetry- breaking, whereas it is well-known that the mobility of a similar homogeneous dimer (conductive or dielectric) is always null under the same (uniform) forcing.

The formulation of the DC electrostatic problem is outlined in Section 2, where the total potential is given by $\phi_{0}\left(\mu ,\upsilon \right)=-\xi +\chi_{0}\left(\mu ,\upsilon \right)$, where $\chi_{0}\left(\mu ,\upsilon \right)$ is given in Equations (3) and (9) satisfying $\partial \phi_{0}\left(\mu ,\upsilon \right)/\partial \upsilon =0$ on υ=±1. Nevertheless, the induced potential ξ on the perfectly conductive sphere (υ=1, zero inner potential) is given following [3] by:

(60) $$\xi \left(\mu ,1\right)=-\phi_{0}\left(\mu ,1\right)-C=\frac{1}{1+\mu^{2}}-{\left(1+\mu^{2}\right)}^{1/2}\int_{0}^{\infty }A_{0}\left(s\right)\frac{tanh\left(s\right)}{s}J_{0}\left(\mu s\right)ds-C,$$

where C is a constant to be determined and the corresponding ξ potential on the unpolarized dielectric (coated) sphere $\left(\upsilon =-1\right)$ can be practically neglected. Charge conservation arguments applied over the surface of the conducting sphere imply that the integral of the potential $\xi \left(\mu \right)$ over its surface vanishes and thus one gets ([53], 6.656.3):

(61) $$C=\frac{1}{2}-2\iint_{0}^{\infty }A_{0}\left(s\right)\frac{tanh\left(s\right)}{s}J_{0}\left(\mu s\right)\frac{\mu d\mu ds}{{\left(1+\mu^{2}\right)}^{3/2}}=\frac{1}{2}-\int_{0}^{\infty }A_{0}\left(s\right)e^{-s}\frac{tanh\left(s\right)}{s}J_{0}\left(\mu s\right)ds.$$

Substituting Equation (9) in Equation (61) and integrating by parts renders an explicit expression for *C*:

(62) $$C=-\frac{1}{2}-\frac{1}{2}\int_{0}^{\infty }\left[ln\left(2\;cosh\left(s\right)\right)+s\left(tanh\left(s\right)-2\right)\right]sinh\left(s\right)e^{-s}ds\;=\frac{1}{4}\left[1+ln\left(2\right)+\frac{1}{4}\zeta \left(2\right)\right]=0.5261,$$

where $\zeta \left(2\right)=\pi^{2}/6$. Numerical integration of the integral in Equation (62) verified the result presented on the right-hand side of Equation (62).

The dimensionless slip velocity generated by the induced-charge electroosmotic (ICEO) flow past the polarizable sphere, can be described by the HS slip velocity [1,2] as ${\vec{v}}_{S}=-\xi \left(\mu ,1\right){\left.\nabla_{\left|\right|}\phi \right|}_{\upsilon =1}$, where $\nabla_{\left|\right|}$ denotes the tangential (to the surface) gradient. Thus, the axial ICEP mobility of the Janus dimer is given by the following integral:

(63) $$U_{z}=-\frac{1}{S_{d}}\int_{0}^{2\pi }\int_{0}^{\infty }\xi \left(\mu ,1\right)\frac{\partial \phi_{0}\left(\mu ,1\right)}{\partial z}h_{\mu }h_{\phi }d\mu d\phi =-\frac{2\pi }{S_{d}}\int_{0}^{\infty }\xi \left(\mu ,1\right)\frac{\partial z}{\partial \mu }\frac{\partial \phi_{0}\left(\mu ,1\right)}{\partial \mu }\frac{h_{\phi }}{h_{\mu }}d\mu ,$$

where *S_d_* represents the surface area of the dimer. Substituting the values of the metric coefficients $h_{\mu }=1/\left(1+\mu^{2}\right),\;h_{\phi }=\mu /\left(1+\mu^{2}\right)$, and $\partial z/\partial \mu =-2\mu /\left(1+\mu^{2}\right)$ in Equation (63), yields for $S_{d}=2\pi$:

(64) $$U_{z}=2\int_{0}^{\infty }\xi \left(\mu ,1\right)\frac{\partial \phi_{0}\left(\mu ,1\right)}{\partial \mu }\frac{\mu^{2}}{{\left(1+\mu^{2}\right)}^{2}}d\mu .$$

In order to analytically evaluate $\phi_{0}\left(\mu ,1\right)$, we again use the asymptotic expression for *A*_0_(*s*), as given by Equation (10) (see also Figure 2a), namely $A_{0}\left(s\right)tanh\left(s\right)/s=s\left(1-2s\right)e^{-s}sinh\left(s\right)/4$, which leads to:

(65) $$\phi_{0}\left(\mu ,1\right)=-\frac{1}{1+\mu^{2}}+\frac{{\left(1+\mu^{2}\right)}^{1/2}}{4}\int_{0}^{\infty }\left(1-2s\right)e^{-s}J_{0}\left(s\mu \right)d\mu =\frac{1}{4}\left[1-\frac{6}{1+\mu^{2}}\right].$$

Finally, by substituting Equation (65) into Equation (64) one finds:

(66) $$U_{z}=6\int_{0}^{\infty }\left[\frac{6}{1+\mu^{2}}-\left(1+4C\right)\right]\frac{\mu^{3}}{{\left(1+\mu^{2}\right)}^{4}}d\mu =\frac{1-2C}{6}=8.7*10^{-3},$$

where *C* is given by Equation (62). Note that the dimensionless phoretic velocity (mobility) of the Janus dimer is in the negative z direction, namely from the conducting (metallic) sphere towards the coated (dielectric) sphere as expected [3].

## 8. Discussion and Summary

In this work we presented an analytical methodology for evaluating the non-linear (quadratic in the applied field) ICEO problem about doubly connected (non-convex) micro/nano polarizable colloids freely suspended in a conducting (electrolyte) fluid. In particular, we chose to analyze the two touching (fused) spheres (dimer) configuration by applying the R-separable tangent-sphere orthogonal coordinate system. The linearized formulation was based on solving both the electrostatic and hydrodynamic (Stokes regime) problems. These two problems are uncoupled due to the ‘weak’ field (standard model) assumption and using the classical (linearized) PNP formulation. In addition, we considered the EDL around the dimer as thin. The ambient electric forcing can be either of a DC or AC nature as well as spatially uniform or non-uniform. In the course of the analysis, we obtained explicit expressions for the linear and angular mobilities of a freely suspended dimer under various electric forcing and also for the electroosmotic ICEO flow field engendered around a stationary dimer by the same forcing. It was demonstrated that the same approach enabled us to consider the EHD flow of a conducting sphere adjacent to a planar wall (electrode) and to analytically resolve the mobility problem of a free Janus dimer composed of two spheres, one conducting and one dielectric (symmetry-breaking).

First, we considered the case of a homogeneous dimer under an AC uniform field directed along the line of centers. The solution of the electrostatic problem was found by solving a mixed (Robin-type) non-homogeneous boundary condition applied on the dimer’s surface in terms of the electric potential and the imposed RC frequency $\Omega_{}$ (6). The solution was determined by solving a non-trivial complex second-order ODE (8). It was further demonstrated that the asymptotic solution of this ODE can indeed serve as a pretty good approximation (see Figure 2), by comparing it both against the known exact solution for the DC case (9) as well as the corresponding numerical solution (obtained by using a 2nd-order central finite-difference scheme). Thus, we may conclude that, at least for the practical range of forcing frequencies (i.e., below the Maxwell–Wagner limit), where $\Omega_{}$ is of the order of unity, the ‘asymptotic’ approximation may be effectively used. One can then obtain for example, an analytic expression for the polarizability (far-field dipole) of a dimer in terms of the Riemann zeta function. The same approach was further used in Section 3 and Section 4 for the corresponding ICEP problem to explicitly find the angular velocities of a dimer under ROT excitation (two orthogonal out-of-phase uniform fields) and the linear mobility when the dimer was exposed to a general axisymmetric travelling wave (non-uniform AC fields). The mobility spectra in both cases were found to be of a Lorentzian type (compact support), exhibiting a maximum value at a prescribed RC frequency.

As far as ICEO and some related hydrodynamic problems, the integral Stokes stream- function formulation has been used in Section 5 to determine the steady (time-averaged) electroosmotic flow field induced around a stationary dimer due to an axisymmetric uniform AC electric field affected by the corresponding HS velocity slip. The velocity components were expressed in the curvilinear ’tangent-sphere’ coordinate system. They decreased with frequency, depended quadratically on the field, and decayed away from the dimer. A similar stream function integral approach was also applied in Section 7 to determine the EHD flow field induced around a spherical colloid lying next to a planar conducting electrode resulting in an explicit solution as depicted in Figure 4. Finally, we provided a new demonstration for a typical symmetry-breaking DC problem involving a Janus dimer configuration composed of one perfectly conducting sphere and the other purely dielectric. Due to the mismatch in material properties between the spheres, it was shown that such a non-homogeneous dimer (in contrast to the homogeneous case) will acquire a finite mobility along the line of centers. The direction of the phoretic mobility (as expected) is always from the metallic towards the dielectric sphere. The above examples demonstrate the versatility of the presented integral formulation for other multi-connected configurations and engineered colloids such as particle interaction and chaining phenomena of spherical colloids.

## Nomenclature

*A_n_*	Complex function forming the integrand solution of the general time-dependent potential, see Equation (2)
*a*	Radius of the sphere
*C*	Constant (Section 7)
*D*	Diffusivity of the symmetric monovalent electrolyte
${\vec{d}}_{eff}$	Effective dipole
*E*_0_	Forcing amplitude of the excitation
*E*(*t*)	Forcing electric field
*E* ^4^	Biharmonic operator
$\hat{e}$	Unit vector
*h*	Metric coefficient
*I*_0_	Modified Bessel function of the first kind
*J*_0_	Bessel function of the first kind
*k*	Wave number
*n*	Normal unit vector
*P_n_*	Legendre polynomial
*R*	Spherical radius
*r*	Polar radius in the x–y plane, see Figure 3
*S_d_*	Surface area of the dimer
*s*	Integration variable, e.g., Equation (2)
*t*	Time
*U*	Velocity component
$\vec{v}$	Velocity vector
*x*	Axial coordinate, see Figure 1
*y*	Lateral coordinate
*z*	The dimer axisymmetric coordinate, see Figure 1
ε	Electric permittivity
ζ	Euler–Riemann zeta function
$\tilde{\eta }$	Spherical angle
η	Dynamic viscosity
Θ°	Angular velocity
$\lambda_{0}$	Nano-metric EDL thickness
$\left(\begin{array}{c} \mu \\ \upsilon \\ \varphi \end{array}\right)$	Orthogonal tangent-sphere coordinate system
ξ	Induced potential
$\vec{\tau }$	Electrostatic torque
ϕ	Total electric potential
$X_{TW}$	Ambient axisymmetric electric forcing of a non-homogenous travelling-wave (TW) excitation (Section 4)
χ	Harmonic function, a component of the total electric potential in the far- field
ψ	Stream function
Ω	RC dimensionless frequency
ω	Frequency
AC	Alternating current
EDL	Electric double layer
DC	Direct current
DEP	Dielectrophoresis
EHD	Electro-hydrodynamic
HS	Helmholtz–Smoluchowski
ICEO	Induced-charge electroosmosis
ICEP	Induced-charge electrophoresis
PNP	Poisson–Nernst–Planck
RC	Resistance–capacitance circuit
ROT	Electro-rotation
TW	Travelling wave
TWDEP	Travelling-wave dielectrophoresis
